# Multidimensional variability in ecological assessments predicts two clusters of suicidal patients

**DOI:** 10.1038/s41598-023-30085-1

**Published:** 2023-03-02

**Authors:** Pablo Bonilla-Escribano, David Ramírez, Enrique Baca-García, Philippe Courtet, Antonio Artés-Rodríguez, Jorge López-Castromán

**Affiliations:** 1 Department of Signal Theory and Communications, Universidad Carlos III de Madrid, Leganés, Spain; 2 Instituto de Investigación Sanitaria Gregorio Marañón, Madrid, Spain; 3 Department of Psychiatry, Centre Hospitalier Universitaire de Nîmes, Nîmes, France; 4 Instituto de Investigación Sanitaria Fundación Jiménez Díaz, Madrid, Spain; 5 Department of Psychiatry, Hospital Universitario Rey Juan Carlos, Móstoles, Madrid Spain; 6 Universidad Autónoma de Madrid, Madrid, Spain; 7 Department of Psychiatry, Hospital Universitario Fundación Jiménez Díaz, Madrid, Spain; 8 Department of Psychiatry, Hospital Universitario Central de Villalba, Madrid, Spain; 9 Department of Psychiatry, Hospital Universitario Infanta Elena, Valdemoro, Madrid Spain; 10 Universidad Católica del Maude, Talca, Chile; 11 CIBERSAM, Instituto de Salud Carlos III, Madrid, Spain; 12 IGF, CNRS-INSERM, Université de Montpellier, Montpellier, France; 13 Department of Emergency Psychiatry and Acute Care, Centre Hospitalier Universitaire de Montpellier, Montpellier, France; 14 Evidence-Based Behavior, Madrid, Spain

**Keywords:** Risk factors, Human behaviour

## Abstract

The variability of suicidal thoughts and other clinical factors during follow-up has emerged as a promising phenotype to identify vulnerable patients through Ecological Momentary Assessment (EMA). In this study, we aimed to (1) identify clusters of clinical variability, and (2) examine the features associated with high variability. We studied a set of 275 adult patients treated for a suicidal crisis in the outpatient and emergency psychiatric departments of five clinical centers across Spain and France. Data included a total of 48,489 answers to 32 EMA questions, as well as baseline and follow-up validated data from clinical assessments. A Gaussian Mixture Model (GMM) was used to cluster the patients according to EMA variability during follow-up along six clinical domains. We then used a random forest algorithm to identify the clinical features that can be used to predict the level of variability. The GMM confirmed that suicidal patients are best clustered in two groups with EMA data: low- and high-variability. The high-variability group showed more instability in all dimensions, particularly in social withdrawal, sleep measures, wish to live, and social support. Both clusters were separated by ten clinical features (AUC = 0.74), including depressive symptoms, cognitive instability, the intensity and frequency of passive suicidal ideation, and the occurrence of clinical events, such as suicide attempts or emergency visits during follow-up. Initiatives to follow up suicidal patients with ecological measures should take into account the existence of a high variability cluster, which could be identified before the follow-up begins.

## Introduction

Implementing secondary prevention methods with suicidal patients is one of the best public health tools we have to actually avert suicidal outcomes^1,2^. Suicidal patients are often assessed in the Emergency Room (ER) or outpatient clinics, and most of them are not hospitalized. Psychiatrists and psychologists modulate clinical care depending on the assessments made in clinical settings, for instance, increasing or reducing the frequency of consultations or adapting the pharmacological treatment, but we have only indirect information about the situation of the patients in their daily life. This situation has changed in the last decade with the widespread diffusion of smartphones, which allows the use of Ecological Momentary Assessment (EMA) to monitor suicidal patients routinely. EMA follow-up could eventually lead to timely Ecological Momentary Interventions (EMI) and boost prevention efforts. A good example was provided by Wang et al. in a prognostic study with 83 inpatients that completed EMA surveys of Suicidal Ideation (SI) several times per day during their hospitalization^3^. The real-time data of SI allowed them to predict with great accuracy the risk of post-discharge suicide attempts. However, EMA studies in suicidology have been neglected until recently^4^ and those that exist are limited in their sample size or duration^5^, mainly due to the fatigue of the patients with the use of the assessment apps and the sensitivity of the questions they answer^6^.

Although ecological studies of suicidal patients focused first on the intensity and duration of suicidal ideas, the finding of sharp changes in very short periods pushed the interest in measuring their variability^7^. One of the first studies in this area used paper-and-pencil methods and asked university students to fill up a daily battery of questionnaires for 4 weeks^7,8^. Their results showed an association between SI variability and a previous history of suicide attempts and suggested that SI variability in multiple attempters was independent of their mood. More recent work, based on EMA, suggests that SI variability could be a marker of risk during the follow-up of suicidal patients^3,9,10^. For instance, the predictive accuracy of Wang et al.’s model, which is described above, was improved with dynamic data on SI changes^3^. In another paper, Oquendo et al. examined 6 weeks of discontinued EMA data of 51 depressed patients and found stable levels of SI variability for each patient over 2 years^9^. They also found that high variability could increase the propensity to experience SI when exposed to stressful life events. In line with this finding, impulsivity traits were associated with SI variability, but not SI intensity, in another EMA study that followed up 84 depressed patients for 10 days^10^.

SI variability may be associated with a particular patient profile characterized by mood or affective instability, which is defined by sudden and recurrent changes in affect. For instance, the level of affective instability predicted SI variability, independently of depression severity, in a sample of female patients with borderline personality disorder^11^. Affective instability has also been associated with peaks of SI in other samples such as working women^12^, and patients suffering from depression and anxiety^13^, bipolar disorder^14^, or psychosis^15^.

In this paper, we have analyzed the data of the Smartcrises study^16^, the largest and longest EMA study with suicidal patients to date. The EMA questions in the Smartcrises study focus on three factors that have been related to the emergence of SI (namely sleep, appetite, and social connectedness), as well as SI and suicidal risk. The choice of sleep, appetite, and social connectedness was based on their potential use as behavioral markers of depression and suicide risk, independently of cultural determinants. Sleep disturbances, particularly insomnia and nightmares, are associated with an increased risk of suicidal behavior (including SI, attempts, and suicide) across diagnoses, cultures, and age groups^17^. Changes in social connectedness caused by acute psychosocial stress, such as the experience of social exclusion, can act as a trigger for suicidal behavior and induce specific biological and psychological changes^18^. Appetite variations have been also associated with depressive symptomatology and suicidal behavior^19,20^.

**Figure 1:**
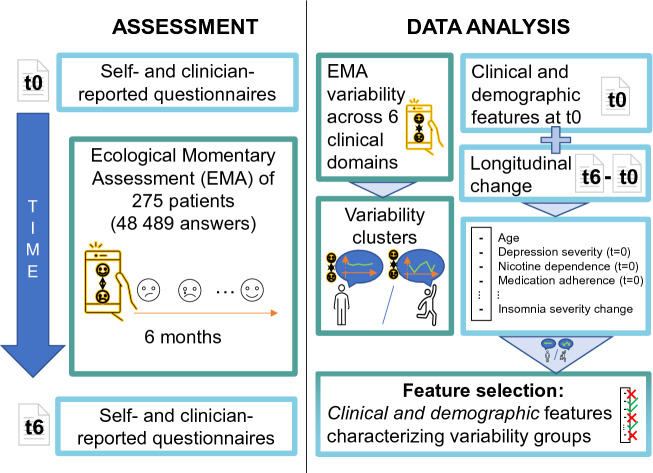
Overview of the study. Notice that the main aim of this paper is to find and analyze which clinical and demographic features could be associated with each *variability* profile. The candidate set of clinical and demographic features (whose possible association to the variability profiles was analyzed) was obtained upon inclusion and at the end of the follow-up. Hence, some longitudinal features were considered by computing the change at those discrete time instances.

The aim of this paper was to study the clinical profile of suicidal patients according to the variability of their EMA responses. Consequently, we clustered them according to EMA variability during the follow-up and then compared the demographic and clinical features between the clusters. We hypothesized that high variability would be associated with features of clinical severity, such as depression severity or clinical events during follow-up. The results could help to improve the design of future EMI for suicide prevention. An overview of the study is provided in Fig. 1.

## Methods

### Sample

This study analyzes a set of 275 out of 419 patients from the *Smartcrises* study^16^ that complies with all of the following requirements: (1) a complete diagnostic assessment with Mini International Neuropsychiatric Interview (MINI) version 7.0.2 is available; (2) the age and gender of the patients are known; and (3) the EMA variability can be computed in at least one domain, i.e. the participant has answered at least to three prompts in a domain (notice that EMA variability and domains are formally defined in the “Statistical analysis” section, see also Details of the patient selection process in Supplementary Material). Compared to included patients, non-included patients were more likely to be older and retired. They also showed smaller decreases in depressive symptomatology measured by the Inventory of Depressive Symptomatology (IDS), from baseline to the end of follow-up, and higher scores of psychological pain at inclusion (P values < 0.05). None of these differences persisted after correction for multiple comparisons. Refer to the Supplementary Material for the details about the patient selection process; for instance, Fig. S3 shows a Venn diagram with numbers and reasons for exclusion.

Patients were recruited from outpatient and emergency psychiatric departments in five clinical centers across Spain and France. EMA was performed with the MeMind Wellness Tracker app (available at the App Store and Google Play) as software, and the participants’ smartphones as hardware. The language of the questionnaires and the mobile application, which included all assessment scales and EMA questions, was adapted to the country. Relevant inclusion criteria include being 18 years old or older, and a clinical assessment due to a suicidal crisis in the last 7 days. Patients diagnosed with a current manic, hypomanic or mixed affective episode, or any lifetime psychotic disorder were excluded. Patients with bipolar disorder could be included only in the depressive phases of the illness and in the absence of any manic or mixed symptomatology. Signed written consent (i.e., informed word) was obtained from all participants. Ethics and privacy regulation, including, but not limited to the Declaration of Helsinki^21^, was followed, and all methods were carried out in accordance with relevant guidelines and regulations. The study protocol was approved by the Institutional Review Board (IRB) of Fundación Jiménez Díaz Hospital, Spain, on the 25th of June 2017 (LSRG-1-005-16), and by the Comité de Protection des Personnes Ouest IV, France, on the 3rd of July 2018 (20187-A02634-49). The study is registered as a clinical trial since October 2018 (ClinicalTrials.gov Identifier: NCT03720730).

### Study design

The *Smartcrises* study combines validated clinical scales with EMA to assess the health condition of the patients. The battery of self-reported and clinician-reported questionnaires was administered in clinical visits upon inclusion and after 6 months at the end of follow-up. EMA questions were presented longitudinally throughout the study and comprise several clinical domains: (1) the assessment of suicide risk; (2) the wish to live/die; the level of social connectedness, measured in two parts: (3) social support and (4) social withdrawal; (5) sleep disturbances; and (6) the level of appetite. Notice that one of the main deterrents of EMA are fatigue effects, which consist in a decrease in the response rate as time goes by due to the repetitiveness of the questions, and this may result in withdrawal from the study^22,23^. To tackle this problem and obtain EMA data beyond 1–2 weeks, both the frequency and the set of asked questions changed during the study. Up to 32 EMA questions were defined. Importantly, questions on wish to live/die, instead of direct questions on SI, were chosen to minimize the potentially adverse effects of repeated prompts over a long period. The wish to live/die captures passive ideas of suicide, the main outcome of the *Smartcrises* study, and is a proxy measure of SI^24,25^. Indeed, passive SI is strongly correlated with active SI and other suicidal outcomes, including suicide death, according to a recent metanalytic review^26^.

The EMA assessment is inspired by the Salzburg Suicide Process (SSP) questionnaire, which was designed to assess dynamic changes in suicidal risk^27^. Most of the questions of the SSP questionnaire are extracted from validated clinical questionnaires. Hence, as shown in Table 1, our EMA assessment compiles questions relevant for a dynamic follow-up of suicidal patients along different domains. Each domain is evaluated with questions inspired from a given validated clinical questionnaire. In particular, EMA questions include items from the Suicidal Status Form (SSF)^28^, the Perceived Social Support Questionnaire (PSSQ)^29^, the Interpersonal Needs Questionnaire (INQ)^30^, the Insomnia Severity Index (ISI)^31^, and the Council of Nutrition Appetite Questionnaire (CNAQ)^32^. However, only one to five of the 32 questions were randomly chosen every day to be presented to the patients. The frequency of the questions was progressively reduced based on the evolution of the suicide risk after an attempt: 4–5 prompts in the first month, 3–4 in the next 2 months and finally 1–2 prompts in the last 3 months^33^. It must be mentioned that the EMA questions were not selected completely at random; instead, those related to sleep and suicide were given much higher probabilities of being asked, due to their importance, and a turn-over system was used in order to avoid repetitions. This approach reduces EMA fatigue, at the expense of every set of responses for the same question being sampled non-equidistantly (non-uniformly).

**Table 1:** EMA questions.

EMA	Question	Domain
1	I feel psychological pain (not including physical pain)	Suicide risk
2	I feel stress (overwhelmed)	
3	I am agitated (restlessness)	
4	I am full of hope	
5	I feel hate or anger toward myself	
6	I feel hate or anger toward other people	
7	My wish to live is	Wish to live
8	My wish to die is	
9	I wish there were a trusted person with whom I could talk about my personal problems	Social support
10	I feel like an outsider	
11	I have the impression that important people around me want to decide what I should think and do	
12	I wish I received more appreciation and affection from other people	
13	I think that I contribute to the well-being of my family/friends	Social withdrawal
14	I think that I contribute to the well-being of the people who are close to me	
15	I feel disconnected from other people	
16	Last night I had problems falling asleep	Sleep
17	Last night I had problems staying asleep	
18	This morning I had problems because I woke up too early	
19	Currently other people think that my sleep problems affect my quality of life	
20	When I woke up I felt	
21	Last night the quality of my sleep was	
22	Today I am satisfied with my quality of sleep	
23	I am currently worried or stressed about my sleep problems	
24	Currently my sleep problems interfere with my daily activity	
25	Today I feel daytime fatigue due to my sleep problems	
26	During the last days my appetite is	Appetite
27	During the last days I feel full after eating	
28	During the last days I feel hungry	
29	During the last days food tastes	
30	Compared to when I was younger, food tastes	
31	During the last days I eat	
32	During the last days I feel sick or nauseated when I eat	

Refer to Supplementary Table S1 for the details of the domains and EMA questions.

### Data management

A total of 48,489 answers to the 32 EMA questions from the 275 patients were analyzed in this study. Those answers were appropriately transformed so that they were all expressed in the range 0 to 100; 100 indicating the worst possible mental condition of the patient, regardless of the fact that some of them were reversed worded^34^. This transformation was done for clarity and displaying purposes to allow visual comparison. Parenthetically, the scores (but not individual items) of the clinical questionnaires were transformed in like manner.

Besides EMA data, information from regular assessments in the *Smartcrises* study includes demographic and clinical data, as well as validated questionnaires. Demographic data comprised: the number of years studying since the beginning of primary school, the employment situation, the marital status, and the country of recruitment. Clinically meaningful events during follow-up (including self-harm, suicide attempts, emergency visits, or hospitalizations in psychiatry that were obtained from clinical records) were computed in a binary variable. Depressive symptoms were assessed with the clinician-reported version of the IDS^35^. The Columbia-suicide severity rating scale (C-SSRS)^36^ was used to assess recent suicidal ideas and lifetime suicidal behavior at inclusion.

In addition, other features were computed from the following questionnaires, which were all self-reported. The overall score of the following scales was obtained: the medication adherence rating scale (MARS)^37^, the CAGE questionnaire for alcohol addiction^38^, the Fagerström Test for Nicotine Dependence (FTND)^39^, the List of Threatening Experiences (LTE)^40^, and the insomnia severity index (ISI)^31^. The dimensions of emotional, physical, and sexual abuse or neglect were obtained from the childhood trauma questionnaire (CTQ)^41^. The 11th version of the Barratt Impulsiveness Scale (BIS)^42^ was used to measure an overall score of impulsivity, as well as the first-order factors of the scale. Further, moral and physical pain were obtained via visual analog scales (VAS)^43^ that were administered upon inclusion. All these instruments were administered at baseline and at the end of follow-up, except the LTE, BIS, VAS, and CTQ, which were used only at the baseline assessment.

The mean of available values for each patient, or ipsative mean^44^, was used to impute the missing values after accounting for reversed-scored questions. This technique is based on the fact that the items in each questionnaire (or its dimension) are related to a single psychometric construct and should be highly correlated. Unlike simply removing the incomplete data (a “complete case analysis”), this technique can help reduce potential biases^45^. Nonetheless, if the missing rate of any questionnaire was over 20% for a given patient, the data was discarded^46^. Score changes, instead of last point values, were used when follow-up questionnaires were available to extract longitudinal features. Finally, marginal diagnoses representing less than 5% of the patients and overall scores of questionnaires with different dimensions were not considered in order to reduce the number of features in the dataset. All data management and statistical analysis were performed using the MATLAB software^47^.

### Statistical analysis

EMA variability was assessed by computing the Median Absolute Deviation (MAD) of the absolute value of the successive slopes^48^. Refer to Supplementary Material, and Fig. S2, for a discussion about the choice of such a metric. Notice that variability was not computed for each individual EMA question directly, since some items (like those related to sleep quality) were intentionally presented more often than others. Thus, the EMA questions were grouped into six domains, namely: suicide risk, wish to live/die, social support, social withdrawal, sleep, and appetite. They correspond to the EMA questions 1 to 6, 7 and 8, 9 to 12, 13 to 15, 16 to 25, and 26 to 32, respectively, as shown in Table 1. Each domain was defined by grouping the EMA questions from a validated clinical questionnaire (for instance, sleep with the ISI or appetite with the CNAQ), with the exception of the two questions on death wish and wish to live. In this way, EMA questions from the same domain were treated as the same question, but expressed differently, and the variability was computed for each domain, containing a larger number of observations, as opposed to analyzing the questions separately. To validate the domains, the Kendall correlation coefficients^49^ of the EMA questions within each domain were computed to make sure that they were positively correlated.

A Gaussian Mixture Model (GMM) was used to cluster the patients based on their multidimensional variabilities (i.e., the EMA variabilities along the six domains). This provided a comprehensive analysis of the variability of the patients, which is not limited to a single clinical aspect (e.g., SI). The GMM’s inference was performed via the Expectation–Maximization (EM) algorithm, which allows to infer missing variability domains by taking the conditional expectations of the missing values given the current parameter estimates and the observed values at every maximization (a.k.a. M) step^50^. The optimal number of groups was determined by the Bayesian Information Criterion (BIC), which produces accurate results even in the inimical scenario of not missing-at-random (NMAR) data^51^. The analysis of the clinical differences between the groups is twofold. On the one hand, phenotypic profiles were compared using the two-sample Student’s t test computing the pooled estimate of the standard deviation for the numeric variables, and the Pearson’s ${\chi }^{2}$ test of homogeneity for the categorical ones. All tests were two-tailed. P values were adjusted for multiple comparisons applying the Holm–Bonferroni method^52^. On the other hand, a forward selection was performed to find the optimal set of clinical features that can be used by a random forest to predict the variability group each patient belongs to.

A random forest is a machine learning algorithm that uses bagging (i.e., a two-step process that stands for “bootstrap aggregating”). In the first or “bootstrap” step, several decision trees^53,54^, a.k.a. weak learners, are trained over different bootstrap samples of the dataset, each one omitting roughly 36.8% of the patients. Omitted patients in each tree are referred to as “out of bag”. In the second or “aggregating” step, the final classification is made taking a democratic (non-weighted) average vote amongst all the trees, thus reducing the risk of overfitting. To further prevent overfitting, diversity (decorrelation among the constituents of the random forest) was enforced by randomly selecting the clinical features that could be used for each decision split^55^. However, some features have significantly more missing data than others (e.g., those measuring follow-up change) and this could bias the forward selection procedure. Hence, surrogate splits were used^56^. In this way, if the optimal feature is missing when classifying the patients, the best surrogate feature will be used to take a decision split and to keep the movement from the root to the leaves of the trees, eventually classifying the patients without discarding partial observations. Since there are both numeric and categorical features, the selected one for each split minimized the P value of the Pearson’s ${\chi }^{2}$ tests of independence between each proposed clinical feature and the variability groups. Unlike the standard Classification and Regression Trees (CART) algorithm, this procedure is not biased toward those features that have many levels, not underestimating the importance of the categorical ones^57^.

The hyperparameters of the random forest were adjusted by employing Bayesian optimization^58^ upon each candidate feature set analyzed during the forward selection. The performance was assessed computing the Area Under the Curve (AUC) of the Receiver Operating Characteristic (ROC) curve^59^ of the predictions of the out of bag patients. Notice that when making predictions for the out of bag patients, only the subset of decision trees that have not been trained using a particular set of patients is used, yielding an unbiased estimator of the true random forest performance that approaches that of leave-one-out cross-validation, but which greatly reduces the computational burden^60^. Details of the statistical methods are provided in the Supplementary Material.

## Results

### Sample description

The study sample of 275 patients had a mean age of 40 years with a Standard Deviation (SD) of 14 years, approximately. It was mainly composed by females (n = 185, 67.27%), where “n” is the number of patients. Almost half of the patients were single (n = 123), and roughly one-third (n = 98) were married or had been cohabiting for more than 6 months. Study participants reported intermediate to high educational levels, with a mean number of years studying since the beginning of primary school of 13 with a SD of 3, approximately. There was a balanced proportion of around one-third of employed and unemployed patients, and patients with an incapacity to work. The main psychiatric diagnoses were post-traumatic stress disorder (n = 74, 26.91%), major depressive disorder (n = 40, 14.55%), and binge eating disorder (n = 23, 8.36%). The base rates of clinically meaningful events during follow-up in the total sample (n = 374) are 6.15% for SI, 1.60% for self-harm, 10.43% for suicide attempts, and 5.08% for emergency hospitalizations.

The participants answered the EMA questions for a mean time period of approximately 130 days with a SD of 104 days. The average length of follow-up was 148 days (SD = 116) in low-variability patients and 98 days (SD = 66) in high-variability patients. The participants answered a mean number of 176 EMA questions with a SD of 161. The mean number of answered questions was 190 (SD = 183) in low-variability patients and 150 (SD = 104) in high-variability patients. The differences between the groups were statistically significant in both cases: *t*_273_ = 3.91, *P* < 0.001 and *t*_273_ = 2.00, *P* = 0.046, respectively. The EMA response percentage was 43.25% for all the patients (maximum: 72.64%, minimum: 16.97%), 45.48% (maximum: 70.53%, minimum: 20.48%) for the low-variability group, and 39.11% (maximum: 83.07%, minimum: 7.64%), for the high-variability group. A detailed description of response rates through follow-up can be found in Fig. S1.

### Validation of the domains

**Figure 2:**
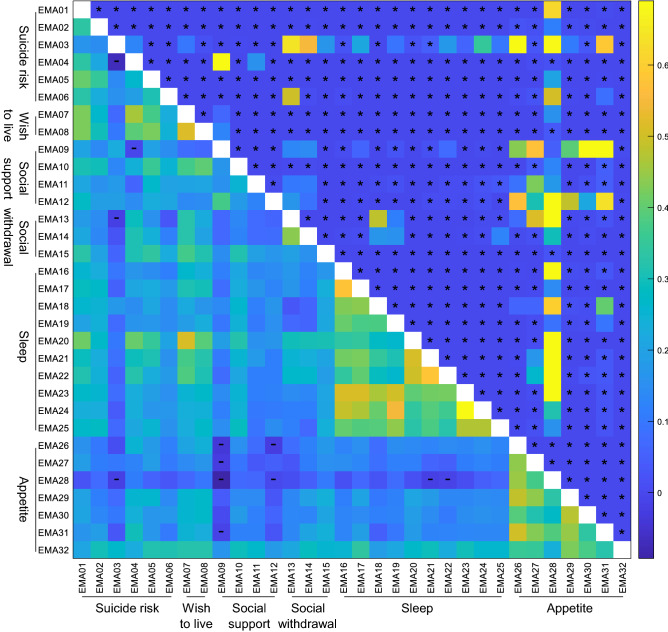
Correlation coefficients among the EMA questions. Correlation of the EMA questions after rescaling them from 0 to 100, where 100 represent worse health condition. Lower triangular portion: Kendall correlation coefficients; “–” for negative values. The main diagonal is intentionally empty for clarity. Upper triangular portion: P values of the hypothesis that the corresponding Kendall correlation value is 0; “*” for P values strictly lower than 0.05. The colormap on the right is used for both Kendall correlation coefficients and P values, and ranges from the overall minimum and maximum values of those quantities.

Figure 2 succinctly shows the correlations among the EMA questions after rescaling them from 0 to 100, accounting for reversed worded questions. There is high correlation amongst them, even if they do not belong to the same domain. The only exception corresponds to the items of the CNAQ assessing appetite that correlate strongly between them but not with items in other domains. Only positive correlations are statistically significant. All questions within the same domain are significantly correlated, with the exception of EMA3, “I am agitated (restlessness)”, which does not correlate with neither EMA4, “I am full of hope”, nor EMA7, “My wish to live is”. Therefore, EMA3 is removed from the “Suicide risk” domain. EMA3 does not correlate with many other questions, but the more independent one is EMA28, “During the last days I feel hungry”, since questions about appetite have the weakest inter-domain correlations.

### Cluster analysis

**Figure 3:**
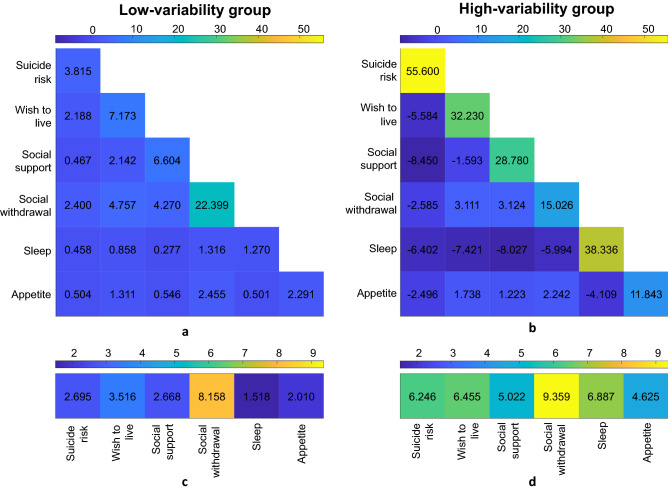
The variability covariances and means of the low-variability group are shown in insets (**a,c**), respectively. The variability covariances and means of the high-variability group are shown in insets (**b,d**), respectively. Recall that they measure *variability*, not absolute values. For simplicity, only the lower triangular and main diagonal region of the variability covariance matrices is shown. Negative covariances indicate that increased variability in one domain is associated with lower variability in the other. The colormaps are the same across the groups to allow visual comparison, and they range from the overall maximum and minimum values of those qualities. Colormaps are shown at the top of each inset.

The BIC determined two variability groups. The first one, or low-variability group, has lower mean variability values and comprises 65% of the sample according to the GMM. The second group, or high-variability group, comprises the rest of the sample. The rest of the parameters are depicted in Fig. 3. Patients from both groups attain the highest mean variability values in the “Social withdrawal” domain. The highest increase in mean variability values when comparing the high- with the low-variability group are found in the “Sleep” and “Suicide risk” domains, with a 4.53- and 2.31-fold increase, respectively. With respect to the covariance in variability (not in mean values), all values are positive in the low-variability group, while there are negative variability interactions in the high-variability group (i.e., variability increases in one domain when the variability of other domain decreases and vice versa) as it happens with the “Sleep” and the rest of the domains. The analysis of the main diagonal elements of Fig. 3a,b reveals that the low-variability group is more homogenous, since it has lower variance in variability for all the domains, especially in “Suicide risk” and “Sleep”.

### Cluster comparison

**Figure 4:**
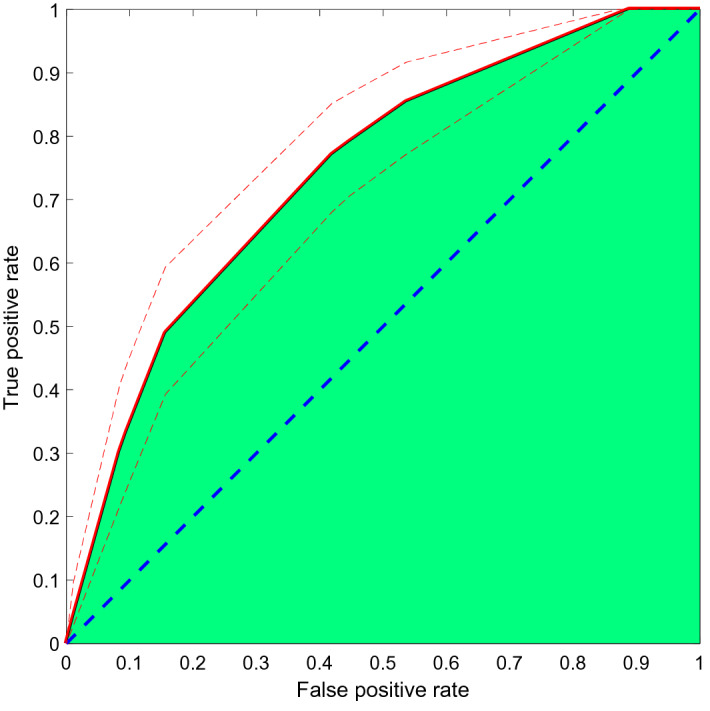
ROC curve. The AUC is 0.74, with 95% CI [0.68, 0.78], estimated by taking 2000 bootstrap samples. Dashed blue line: random guessing reference. Solid red line: mean ROC curve of the automatic prediction of the high-variability group using the selected random forest and clinical features. Green area: AUC. Dashed red lines: 95% confidence intervals of the ROC curve.

**Figure 5:**
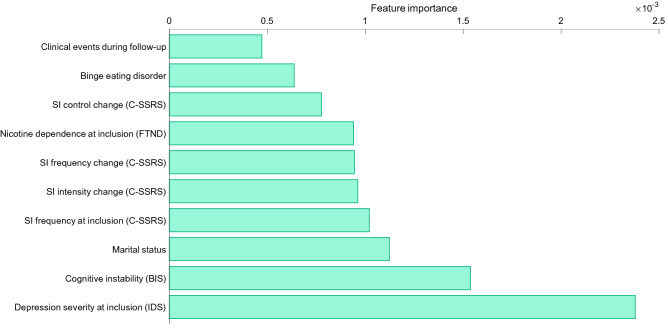
Importance of the ten clinical and demographic features used by the random forest to automatically discriminate patients in the low- and high-variability groups. For clarity, the y-axis is sorted in increasing importance order according to the random forest. The importance is computed by summing all changes in the impurity of the nodes from the parent to the two children thanks to a given clinical feature and its corresponding surrogate splits. Impurity is a measure of how the decisions of a node can separate patients in the low- and high-variability groups and it is measured by the Gini’s diversity index. The sum of impurity changes is normalized by the number of branch nodes.

Table 2 shows the phenotypic profiles of the two variability groups, where no statistical difference is found after correcting for multiple comparisons. Figure 4 illustrates the ROC curve of the automatic classification of the high-variability from the low-variability group, using a random forest. The AUC is 0.74, with 95% Confidence Interval (CI) [0.68, 0.78], which is considered acceptable^61^. The hyperparameters obtained by the Bayesian optimization are listed in Table S2 in the Supplementary Material, and the importance of the ten clinical features chosen by the forward selection to make the classification with the random forest are shown in Fig. 5. According to this selection, the most relevant features to separate the groups (i.e., the features that are most useful for the random forest classification) are the depressive symptoms upon inclusion (IDS), followed by the impulsivity factor of cognitive instability (BIS), the marital status, and the frequency of SI at inclusion (C-SSRS), as well as the change in SI frequency, intensity and control from baseline to follow-up and other clinical factors such as nicotine dependence, binge eating disorders, or the occurrence of clinically meaningful events during follow-up. Single marital status and clinically meaningful events during follow-up were more common in the high-variability group, and all the other features showed also higher scores or higher prevalence in that group (Table 2).

**Table 2:** Demographics and clinical features by cluster according to variability level.

Demographics and clinical features	Low variability group			High variability group			Nominal P value^b^
	Number (% in group)^a^	Mean	SD	Number (% in group)^a^	Mean	SD	
Age	179	41.59	13.55	96	37.43	15.00	**0.020**
Female gender	117 (65.36)			68 (70.83)			0.36
Country of recruitment							
Spain	149 (83.24)			79 (82.29)			0.84
France	30 (16.76)			17 (17.71)			
Marital status							
Married/cohabitation > 6 months	69 (38.76)			29 (30.53)			**0.0047**
Separated/divorced	37 (20.79)			9 (9.47)			
Widowed	5 (2.81)			1 (1.05)			
Single	67 (37.64)			56 (58.95)			
Years of study	165	12.88	3.51	86	12.57	3.37	0.49
Employment situation							
Incapacity	58 (32.77)			26 (27.37)			0.15
Unemployed	52 (29.38)			41 (43.16)			
Retired	8 (4.52)			4 (4.21)			
Employed	59 (33.33)			24 (25.26)			
Psychiatric diagnoses (MINI)^c^							
Agoraphobia	8 (4.47)			10 (10.42)			0.057
Alcohol use disorder^d^	6 (3.35)			3 (3.13)			0.92
Binge eating disorder	9 (5.03)			14 (14.58)			**0.0064**
Bipolar disorder^d^	5 (2.79)			1 (1.04)			0.34
Major depressive disorder	30 (16.76)			10 (10.42)			0.15
Obsessive–compulsive disorder^d^	5 (2.79)			2 (2.08)			0.72
Panic disorder	16 (8.94)			10 (10.42)			0.69
Posttraumatic stress disorder	39 (21.79)			35 (36.46)			**0.0089**
Social anxiety disorder^d^	4 (2.23)			0 (0.00)			0.14
Substance use disorder (non-alcohol)^d^	3 (1.68)			2 (2.08)			0.81
Depression severity at inclusion (IDS)	172	34.53	14.44	89	38.98	16.66	**0.026**
Depression severity change (IDS)	125	− 4.68	11.28	55	− 6.41	12.68	0.36
Suicidal ideation rating (C-SSRS)							
At inclusion							
Controllability	118	2.77	1.83	66	3.24	1.54	0.079
Deterrents	114	2.20	1.60	65	2.26	1.61	0.81
Duration	115	2.90	1.40	63	3.10	1.44	0.39
Frequency	118	3.08	1.37	63	3.33	1.51	0.26
Intensity	122	3.37	1.45	65	3.34	1.46	0.89
Reasons	119	4.03	1.48	67	3.81	1.67	0.36
Change from baseline to end of follow-up							
Controllability	53	0.23	2.09	31	− 0.23	1.50	0.29
Deterrents	49	− 0.16	1.69	33	− 0.61	1.87	0.27
Duration	50	− 0.44	1.61	31	− 0.26	2.05	0.66
Frequency	52	− 0.33	1.13	29	− 0.59	1.96	0.45
Intensity	59	− 0.61	1.49	31	− 0.48	1.48	0.70
Reasons	52	0.35	1.49	33	0.21	2.00	0.72
Number of lifetime suicide attempts	178	2.18	4.18	96	1.80	1.77	0.40
Psychological pain at inclusion	146	60.96	29.11	87	62.53	27.80	0.69
Physical pain at inclusion	145	46.97	26.80	83	44.94	30.58	0.60
Alcohol misuse screening at inclusion (CAGE)	137	16.79	30.64	74	15.54	27.97	0.77
Alcohol misuse screening change (CAGE)	85	− 2.06	24.16	37	− 2.03	27.88	0.99
Childhood trauma (CTQ)							
Emotional abuse	134	44.54	30.49	74	42.97	31.34	0.73
Emotional neglect	139	41.56	26.56	77	41.90	25.53	0.93
Physical neglect	131	18.32	18.19	73	19.35	21.16	0.72
Physical abuse	129	20.78	25.62	70	19.98	26.40	0.83
Sexual abuse	133	19.70	29.96	73	20.02	25.95	0.94
Score of denial	164	13.82	23.03	95	9.82	19.37	0.16
Overall score^d^	127	22.42	15.24	71	22.69	16.56	0.91
Nicotine dependence at inclusion (FTND)	130	17.95	27.10	70	20.94	29.93	0.47
Nicotine dependence change (FTND)	77	− 1.93	16.11	34	− 7.78	20.21	0.11
Impulsivity levels (BIS)							
Attention	140	48.63	21.92	78	51.88	21.82	0.29
Cognitive complexity	132	51.09	17.46	74	49.68	19.10	0.59
Cognitive instability	135	48.97	23.56	67	51.58	24.13	0.46
Motor	132	39.12	20.87	74	42.34	18.44	0.27
Perseverance	127	33.01	15.48	62	35.89	19.00	0.27
Self-control impulsivity	138	42.38	21.26	74	47.88	21.74	0.08
Overall score^d^	136	43.45	13.31	72	46.25	13.38	0.15
Insomnia severity at inclusion (ISI)	137	46.53	22.30	80	49.76	24.21	0.32
Insomnia severity change (ISI)	86	− 7.64	24.04	41	− 9.63	26.42	0.67
Life events at inclusion (LTE)	138	27.84	19.79	78	23.18	20.49	0.10
Medication adherence at inclusion (MARS)	138	34.02	19.16	79	39.66	19.51	**0.039**
Medication adherence change (MARS)	86	0.82	20.48	40	− 6.01	23.51	0.10
Clinically meaningful events during follow-up	38 (22.22)			29 (31.52)			0.10

^a^The sum of the number of patients in the two groups may not add up to 275 due to missing data.

^b^Since no statistical difference is found after correcting for multiple comparisons, only the nominal P values are shown.

^c^Diagnoses representing < 1% of the sample are not shown.

^d^Items shown for completeness, but not considered neither for the classification with random forest nor for the multiple comparison correction.

Nominal P values strictly smaller than 0.05 are marked in bold.

## Discussion

EMA and, by extension, EMI are very promising methods that could transform the field of suicide prevention. In this paper, we have analyzed a large EMA sample of suicidal patients to ascertain if symptom variability was associated with a higher risk of suicidal behavior and/or with other features of clinical severity. Symptom variability defined two clear-cut groups. The high-variability group is characterized by frequent changes not only in SI as previously reported, but also in domains such as social withdrawal, social support, sleep, or appetite. Furthermore, although conventional statistics found few differences between the groups when comparing cross-sectional data (Table 2), machine learning methods allowed us to obtain a good classification performance in this complex dataset with a large number of explanatory variables. Building on prior studies^3,9^, clinical variables such as the severity of depressive symptoms or the frequency of SI separate patients with high and low symptom variability. Importantly, although the occurrence of clinically meaningful events during follow-up, such as suicide attempts or hospitalizations, was not different between the groups according to standard statistics (p = 0.10), those events were almost 10 percentual points more frequent in the high-variability group and the variable was selected by random forest amongst the best features to differentiate high-variability patients.

Extant EMA studies with suicidal patients have shown so far that: (1) SI fluctuates widely, and (2) some clinical features, such as negative affect and disturbed sleep, could be used to predict SI fluctuations in the short-term^5^. However, fluctuations are not restricted to SI and sleep. In our study, social withdrawal was the clinical factor that showed the largest variability in both groups, a finding that could be related to the social sensitivity of suicidal patients. Among low-variability patients, social withdrawal was the only dimension with substantial variability, suggesting that in that group mood variations are mainly externalized through social interactions. Suicidal patients are sensitive to social cues and tend to interpret them negatively^62^, which may lead the patients to minimize their social interactions. An example of this sensitivity can be found in a recent study in which EMA-measured psychological pain correlated with orbitofrontal activation during a social exclusion paradigm in suicide attempters, but not in affective controls^18^. In the same vein, a small EMA study found that SI variability correlated strongly with SI intensity, but also with the intensity and variability of depressive symptoms and changes in social connectedness^63^. The pattern of variability extends thus well beyond SI and the instability of highly variable patients affects their social interactions, sleep and appetite, which is consistent with the fact that almost all EMA items correlated significantly with each other across clinical dimensions (Fig. 2). Interestingly, when high-variability patients fluctuate in one clinical dimension (such as suicide risk or sleep), they can be more stable in other dimensions. This is reflected by the negative correlations in Fig. 3 and suggests that some changes occur sequentially, rather than simultaneously, in the high-variability group. In contrast, fluctuations in the low-variability group tended to occur at the same time across the six clinical dimensions.

Some of the features that were selected by the machine learning algorithm to separate high- and low-variability groups have been related to impulsivity. They include one first-order factor of the BIS (i.e., cognitive instability) which reflects intruding and racing thoughts^42^, but also nicotine dependence^64^, the diagnosis of binge eating disorder^65^, and being single^66^. Since sleep disturbances predict the onset of SI^67^, it is interesting to note that the construct of cognitive instability has been recently identified as a transdiagnostic symptom associated with insomnia severity^68^. The role of impulsivity may also be related to affective instability since they are closely related, and partially overlapping, constructs^69^. A recent study associated SI variability with affective instability in Borderline Personality Disorder (BPD)^11^. We could not verify if BPD was overrepresented in the high-variability cluster because the disorder is not included in the MINI diagnostic assessment, but since childhood trauma and gender did not separate the clusters, the possibility of BPD diagnoses being concentrated in one of them is unlikely. We also lacked data on Attention Deficit and Hyperactivity Disorder (ADHD), another diagnosis that is frequently associated with cognitive impulsivity and emotional dysregulation. Impulsive reactions may also explain why high-variability participants stopped their follow-up earlier, having answered fewer EMA questions, than low-variability ones. Importantly, the relationship between SI and impulsivity is accentuated during suicidal crises^70^ but impulsivity can be the target of both pharmacological and psychotherapeutic interventions.

This study is based on a large sample of suicidal patients that were followed for several months using EMA methods. It is partially limited by missing follow-up data. According to a recent systematic review, the compliance in our sample was in the low range of EMA studies with suicidal patients (44 to 90%), but this could be expected given the long follow-up (prior studies ranging from 4 to 60 days) and the decline of compliance rates over time^71^. The use of a turnover pool of questions and a decreasing number of prompts through the follow-up seems to have reduced fatigue effects since most participants were still responding EMA questions after 4 months. Traditional methods are biased toward features that have fewer missing values. The methods applied in this study, like the EM algorithm used for the inference of the GMM, or the surrogate splits of the random forest were implemented to mitigate such a bias by exploiting the inherent structure of the data. This potential bias has also been tackled by following an agnostic approach for the feature selection, in which the algorithm was free to choose the set of features used for the classification.

A second limitation concerns the complexity of the database, which included a large number of variables and time-lagged information. We used a potent classifier based on random forest methods since off-the-shelf methods failed to provide meaningful results in this high-dimensional dataset with NMAR and correlated data. For example, in EMA, high variability patients respond more at the beginning of the follow-up, but drop out earlier than low-variability ones (Fig. S1). The surrogate splits of the random forest leveraged the natural correlations of the features to deal with the missing values, making those correlations beneficial, and the random feature selection and boosting procedure aimed to obtain robust results and to prevent overfitting^72^. Further, the random forest approach provides, as a byproduct, an objective tool to automatically identify patients with high variability only using demographic and clinical information, providing a probability estimate of the group each patient belongs to. The ability of the classifier to distinguish between groups was good, attaining an AUC of 0.74. Finally, two points should be noted regarding the clinical severity of the sample. First, some patients were recruited after being discharged from the hospital in outpatient consultations, which could select less severely suicidal patients. Second, although the patients excluded from the analyses were fairly similar to those included, higher basal depressive symptomatology and psychological pain suggest that they could represent more severe cases.

In summary, approximately one third of suicidal patients present high SI variability and a general pattern of instability in several domains during their follow-up, which is generally shorter. This pattern can be easily detected in early stages by assessing the severity of SI and depression, as well as impulsivity traits and other factors, and it might be associated with a higher risk of clinical events during follow-up. EMA protocols should be adapted to optimize suicidal risk assessment and preventive interventions in high- and low-variability groups.

## Supplementary Information

Supplementary Information.

## Data Availability

Data is available upon request from the corresponding author.

## References

[1] Zalsman G (2016). Suicide prevention strategies revisited: 10-year systematic review. Lancet Psychiatry.

[2] Turecki G (2019). Suicide and suicide risk. Nat. Rev. Dis. Primer.

[3] Wang SB (2021). A pilot study using frequent inpatient assessments of suicidal thinking to predict short-term postdischarge suicidal behavior. JAMA Netw. Open.

[4] Davidson CL, Anestis MD, Gutierrez PM (2017). Ecological momentary assessment is a neglected methodology in suicidology. Arch. Suicide Res..

[5] Sedano-Capdevila A, Porras-Segovia A, Bello HJ, Baca-García E, Barrigon ML (2021). Use of ecological momentary assessment to study suicidal thoughts and behavior: A systematic review. Curr. Psychiatry Rep..

[6] Porras-Segovia A (2020). Smartphone-based ecological momentary assessment (EMA) in psychiatric patients and student controls: A real-world feasibility study. J. Affect. Disord..

[7] Witte T, Fitzpatrick K, Joinerjr T, Schmidt N (2005). Variability in suicidal ideation: A better predictor of suicide attempts than intensity or duration of ideation?. J. Affect. Disord..

[8] Witte TK, Fitzpatrick KK, Warren KL, Schatschneider C, Schmidt NB (2006). Naturalistic evaluation of suicidal ideation: Variability and relation to attempt status. Behav. Res. Ther..

[9] Oquendo MA (2020). Highly variable suicidal ideation: A phenotypic marker for stress induced suicide risk. Mol. Psychiatry..

[10] Hadzic A (2020). The association of trait impulsivity and suicidal ideation and its fluctuation in the context of the interpersonal theory of suicide. Compr. Psychiatry.

[11] Rizk MM (2019). Variability in suicidal ideation is associated with affective instability in suicide attempters with borderline personality disorder. Psychiatry.

[12] Tian L, Yang Y, Yang H, Huebner ES (2017). Prevalence of suicidal ideation and its association with positive affect in working women: A day reconstruction study. Front. Psychol..

[13] Bowen R, Balbuena L, Peters EM, Leuschen-Mewis C, Baetz M (2015). The relationship between mood instability and suicidal thoughts. Arch. Suicide Res..

[14] Ducasse D (2017). Affect lability predicts occurrence of suicidal ideation in bipolar patients: A two-year prospective study. Acta Psychiatr. Scand..

[15] Palmier-Claus JE (2013). Affective instability prior to and after thoughts about self-injury in individuals with and at-risk of psychosis: A mobile phone based study. Arch. Suicide Res..

[16] Berrouiguet S (2019). Combining mobile-health (mHealth) and artificial intelligence (AI) methods to avoid suicide attempts: The Smartcrises study protocol. BMC Psychiatry.

[17] Lopez-Castroman J, Jaussent I, Baca-Garcia E (2020). Sleep disturbances and suicidal behavior. Behavioral Neurobiology of Suicide and Self Harm.

[18] Olié E (2021). Prefrontal activity during experimental ostracism and daily psychache in suicide attempters. J. Affect. Disord..

[19] van Velzen LS (2022). Risk factors for suicide attempt during outpatient care in adolescents with severe and complex depression. Crisis..

[20] Trivedi MH (2011). Concise health risk tracking scale: A brief self-report and clinician rating of suicidal risk. J. Clin. Psychiatry.

[21] World Medical Association (2001). World Medical Association Declaration of Helsinki. Ethical principles for medical research involving human subjects. Bull. World Health Organ..

[22] Moitra E, Gaudiano BA, Davis CH, Ben-Zeev D (2017). Feasibility and acceptability of post-hospitalization ecological momentary assessment in patients with psychotic-spectrum disorders. Compr. Psychiatry.

[23] Glenn CR (2022). Feasibility and acceptability of ecological momentary assessment with high-risk suicidal adolescents following acute psychiatric care. J. Clin. Child Adolesc. Psychol..

[24] Baca-Garcia E (2011). Estimating risk for suicide attempt: Are we asking the right questions?: Passive suicidal ideation as a marker for suicidal behavior. J. Affect. Disord..

[25] Porras-Segovia A (2021). Disturbed sleep as a clinical marker of wish to die: A smartphone monitoring study over three months of observation. J. Affect. Disord..

[26] Liu RT, Bettis AH, Burke TA (2020). Characterizing the phenomenology of passive suicidal ideation: A meta-analysis of its prevalence, psychiatric comorbidity, correlates, and comparisons with active suicidal ideation. Psychol. Med..

[27] Fartacek C, Schiepek G, Kunrath S, Fartacek R, Plöderl M (2016). Real-time monitoring of non-linear suicidal dynamics: Methodology and a demonstrative case report. Front. Psychol..

[28] Jobes DA, Linehan MM (2016). Managing Suicidal Risk: A Collaborative Approach.

[29] Zimet GD, Dahlem NW, Zimet SG, Farley GK (1988). The multidimensional scale of perceived social support. J. Pers. Assess..

[30] Van Orden KA, Cukrowicz KC, Witte TK, Joiner TE (2012). Thwarted belongingness and perceived burdensomeness: Construct validity and psychometric properties of the interpersonal needs questionnaire. Psychol. Assess..

[31] Bastien CH, Vallières A, Morin CM (2001). Validation of the insomnia severity index as an outcome measure for insomnia research. Sleep Med..

[32] Wilson M-MG (2005). Appetite assessment: Simple appetite questionnaire predicts weight loss in community-dwelling adults and nursing home residents. Am. J. Clin. Nutr..

[33] Cedereke M, Öjehagen A (2005). Prediction of repeated parasuicide after 1–12 months. Eur. Psychiatry.

[34] Zhang X, Noor R, Savalei V (2016). Examining the effect of reverse worded items on the factor structure of the need for cognition scale. PLoS ONE.

[35] Rush AJ, Carmody T, Reimitz P-E (2000). The inventory of depressive symptomatology (IDS): Clinician (IDS-C) and self-report (IDS-SR) ratings of depressive symptoms. Int. J. Methods Psychiatr. Res..

[36] Posner K (2011). The Columbia-suicide severity rating scale: Initial validity and internal consistency findings from three multisite studies with adolescents and adults. Am. J. Psychiatry.

[37] Fialko L (2008). A large-scale validation study of the medication adherence rating scale (MARS). Schizophr. Res..

[38] Ewing JA (1984). Detecting alcoholism: The CAGE questionnaire. JAMA.

[39] Meneses-Gaya IC, Zuardi AW, Loureiro SR, de Crippa JAS (2009). Psychometric properties of the Fagerström test for nicotine dependence. J. Bras. Pneumol..

[40] Brugha TS, Cragg D (1990). The list of threatening experiences: The reliability and validity of a brief life events questionnaire. Acta Psychiatr. Scand..

[41] Bernstein DP (1994). Initial reliability and validity of a new retrospective measure of child abuse and neglect. Am. J. Psychiatry.

[42] Patton JH, Stanford MS, Barratt ES (1995). Factor structure of the Barratt Impulsiveness Scale. J. Clin. Psychol..

[43] Reips U-D, Funke F (2008). Interval-level measurement with visual analogue scales in internet-based research: VAS Generator. Behav. Res. Methods.

[44] Shrive FM, Stuart H, Quan H, Ghali WA (2006). Dealing with missing data in a multi-question depression scale: A comparison of imputation methods. BMC Med. Res. Methodol..

[45] Bono C, Ried LD, Kimberlin C, Vogel B (2007). Missing data on the center for epidemiologic studies depression scale: A comparison of 4 imputation techniques. Res. Soc. Adm. Pharm..

[46] Imai H (2014). Ipsative imputation for a 15-item geriatric depression scale in community-dwelling elderly people. Psychogeriatrics.

[47] MATLAB (2019). Version 9.7.0.1319299 (R2019b) Update 5.

[48] Bonilla-Escribano P, Ramírez D, Porras-Segovia A, Artés-Rodríguez A (2021). Assessment of variability in irregularly sampled time series: Applications to mental healthcare. Mathematics.

[49] Puth M-T, Neuhäuser M, Ruxton GD (2015). Effective use of Spearman’s and Kendall’s correlation coefficients for association between two measured traits. Anim. Behav..

[50] Murphy KP, Murphy KP (2012). Mixture models and the EM algorithm. Machine Learning: A Probabilistic Perspective.

[51] Ibrahim JG, Zhu H, Tang N (2008). Model selection criteria for missing-data problems using the EM algorithm. J. Am. Stat. Assoc..

[52] Wright SP (1992). Adjusted p-values for simultaneous inference. Biometrics.

[53] Delgado-Gómez D, Baca-García E, Aguado D, Courtet P, López-Castromán J (2016). Computerized adaptive test vs decision trees: Development of a support decision system to identify suicidal behavior. J. Affect. Disord..

[54] Delgado-Gómez D, Laria JC, Ruiz-Hernández D (2019). Computerized adaptive test and decision trees: A unifying approach. Expert Syst. Appl..

[55] Breiman L (2001). Random forests. Mach. Learn..

[56] Springer, C. & Kegelmeyer, W. P. Feature selection via decision tree surrogate splits. In *2008 19th International Conference on Pattern Recognition* 1–5. 10.1109/ICPR.2008.4761257 (2008).

[57] Loh, W.-Y. & Shih, Y.-S. *Split Selection Methods for Classification Trees* 26 (1997).

[58] Gao L, Ding Y (2020). Disease prediction via Bayesian hyperparameter optimization and ensemble learning. BMC Res. Notes.

[59] Provost F, Fawcett T (2001). Robust classification for imprecise environments. Mach. Learn..

[60] James G, Witten D, Hastie T, Tibshirani R, James G, Witten D, Hastie T, Tibshirani R (2013). Tree-based methods. An Introduction to Statistical Learning.

[61] Hosmer DW, Lemeshow S, Sturdivant RX, Hosmer DW, Lemeshow S, Sturdivant RX (2013). Assessing the fit of the model. Applied Logistic Regression.

[62] Hagen J, Knizek BL, Hjelmeland H (2017). Mental health nurses’ experiences of caring for suicidal patients in psychiatric wards: An emotional endeavor. Arch. Psychiatr. Nurs..

[63] Peters EM (2020). Instability of suicidal ideation in patients hospitalized for depression: An exploratory study using smartphone ecological momentary assessment. Arch. Suicide Res..

[64] Martinez S (2021). The acute and repeated effects of cigarette smoking and smoking-related cues on impulsivity. Drug Alcohol Rev..

[65] Waltmann M, Herzog N, Horstmann A, Deserno L (2021). Loss of control over eating: A systematic review of task based research into impulsive and compulsive processes in binge eating. Neurosci. Biobehav. Rev..

[66] Lim M, Lee S, Park J-I (2016). Differences between impulsive and non-impulsive suicide attempts among individuals treated in emergency rooms of South Korea. Psychiatry Investig..

[67] Chu C, Nota JA, Silverman AL, Beard C, Björgvinsson T (2019). Pathways among sleep onset latency, relationship functioning, and negative affect differentiate patients with suicide attempt history from patients with suicidal ideation. Psychiatry Res..

[68] Weiner L (2021). Investigating racing thoughts in insomnia: A neglected piece of the mood-sleep puzzle?. Compr. Psychiatry.

[69] Peters EM, Baetz M, Marwaha S, Balbuena L, Bowen R (2016). Affective instability and impulsivity predict nonsuicidal self-injury in the general population: A longitudinal analysis. Borderline Personal. Disord. Emot. Dysregul..

[70] Liu RT, Trout ZM, Hernandez EM, Cheek SM, Gerlus N (2017). A behavioral and cognitive neuroscience perspective on impulsivity, suicide, and non-suicidal self-injury: Meta-analysis and recommendations for future research. Neurosci. Biobehav. Rev..

[71] Kivelä L, van der Does WAJ, Riese H, Antypa N (2022). Don’t miss the moment: A systematic review of ecological momentary assessment in suicide research. Front. Digit. Health.

[72] Altman N, Krzywinski M (2017). Ensemble methods: Bagging and random forests. Nat. Methods.

