# Supplementary material for: Multidimensional variability in ecological assessments predicts two clusters of suicidal patients
Source: Sci Rep. 2023 Mar 2;13:3546. doi: 10.1038/s41598-023-30085-1 (PMC9981613; doi:10.1038/s41598-023-30085-1)
Supplement: Supplementary file 1 — Supplementary Information. [file 41598_2023_30085_MOESM1_ESM.docx]

# Supplementary materials

## Details of the domains and EMA questions

**Table S1.** EMA questions and domains.

| **EMA** | **Question^a^** | **Type^b^** | **Domain** | **Source^c^** |
| --- | --- | --- | --- | --- |
| 1 | I feel psychological pain (not including physical pain) | S1 | Suicide risk | SSF |
| 2 | I feel stress (overwhelmed) | S2 |  |  |
| 3 | I am agitated (restlessness) | S3 |  |  |
| 4 | I am full of hope | S4 |  |  |
| 5 | I feel hate or anger toward myself | S5 |  |  |
| 6 | I feel hate or anger toward other people | S5 |  |  |
| 7 | My wish to live is | S6 | Wish to live |  |
| 8 | My wish to die is | S7 |  |  |
| 9 | I wish there were a trusted person with whom I could talk about my personal problems | S8 | Social support | PSSQ |
| 10 | I feel like an outsider | S8 |  |  |
| 11 | I have the impression that important people around me want to decide what I should think and do | S8 |  |  |
| 12 | I wish I received more appreciation and affection from other people | S8 |  |  |
| 13 | I think that I contribute to the well-being of my family/friends | S8 | Social withdrawal | INQ |
| 14 | I think that I contribute to the well-being of the people who are close to me | S8 |  |  |
| 15 | I feel disconnected from other people | S8 |  |  |
| 16 | Last night I had problems falling asleep | C1 | Sleep | ISI |
| 17 | Last night I had problems staying asleep | C1 |  |  |
| 18 | This morning I had problems because I woke up too early | C1 |  |  |
| 19 | Currently other people think that my sleep problems affect my quality of life | S8 |  |  |
| 20 | When I woke up I felt | S9 |  |  |
| 21 | Last night the quality of my sleep was | S9 |  |  |
| 22 | Today I am satisfied with my quality of sleep | S10 |  |  |
| 23 | I am currently worried or stressed about my sleep problems | C2 |  |  |
| 24 | Currently my sleep problems interfere with my daily activity | C2 |  |  |
| 25 | Today I feel daytime fatigue due to my sleep problems | S8 |  |  |
| 26 | During the last days my appetite is | C3 | Appetite | CNAQ |
| 27 | During the last days I feel full after eating | C4 |  |  |
| 28 | During the last days I feel hungry | C5 |  |  |
| 29 | During the last days food tastes | C6 |  |  |
| 30 | Compared to when I was younger, food tastes | C7 |  |  |
| 31 | During the last days I eat | C8 |  |  |
| 32 | During the last days I feel sick or nauseated when I eat | C9 |  |  |

^a^EMA questions 1 to 3, 5, 6, 8 to 12 ,15 to 19, and 23 to 25 were reversed worded.

^b^Overall, 20 and 12 of the questions were answered by means of a slider and by a 5-point Likert scale, respectively. Abbreviations for the type of response: “S” stands for an integer-precision slider from 0 to 100, they are enumerated depending on the associated meaning of the extreme values which was shown to the patients. S1: No pain, maximum pain; S2: No stress, maximum stress; S3: No agitation, maximum agitation; S4: No hope, maximum hope; S5: No hate, maximum hate, S6: No wish to live, maximum wish to live; S7: No wish to die, maximum wish to die; S8: Not at all, absolutely; S9: Bad, good; S10: Very unsatisfied, very satisfied. “C” stands for categorical responses, and they are enumerated depending on the set of possible values shown to the patients. C1: None, mild, moderate, severe, extreme; C2: Not at all, a little, to some degree, much, very much; C3: Very small, small, normal, good, very good; C4: Just a few bites, about a third of the meal, more than half of the meal, most of the meal, almost never; C5: Never, occasionally, sometimes, most of the time, all time; C6: Very bad, bad, normal, good, very good; C7: Much worse, worse, just as well, better, much better; C8: Less than one meal a day, one meal a day, two meals a day, three meals a day, more than three meals a day (including snacks); C9: Most of the time, often, sometimes, occasionally, never.

cAbbreviations for the source of the questions: SSF: Suicidal Status Form; PSSQ: Perceived Social Support Questionnaire; INQ: Interpersonal Needs Questionnaire, ISI: Insomnia Severity Index, CNAQ: Council of Nutrition Appetite Questionnaire.

## Details of the response rate of the EMA questions

**Figure S1.** Time-dependent response percentage of the EMA questions among patient groups.

## Details of the statistical methods

#### Choice of the variability metric

Before we considered this study, we performed a comprehensive analysis of the variability metrics that can be used for this type of temporal time series^1^. In that work, we created synthetic data, and we measured how the performance of the different variability metrics was affected as some data points were removed (in other words, we modeled missing data), or the respondent inconsistency increased. We considered more than 945 scenarios, and we also performed some experiments using real data.

We concluded that the most robust and interpretable metric is the Median Absolute Deviation (MAD) of the absolute value of the successive slopes. In other words, we first compute the absolute value of the slopes that the EMA time series describes, and we compute the MAD over that result. Notice that this method supersedes the state-of-the-art approaches, since it directly takes into account the amount of missing data among the samples when the variability is measured, as it works with the slopes of the series, not with the original values. We illustrate this idea in Figure S2.

**Figure S2.** Illustration of the slope method. Notice that the first and third, and second and fourth observations have the same value, but the value of the slopes are different due to different amounts of missing data.


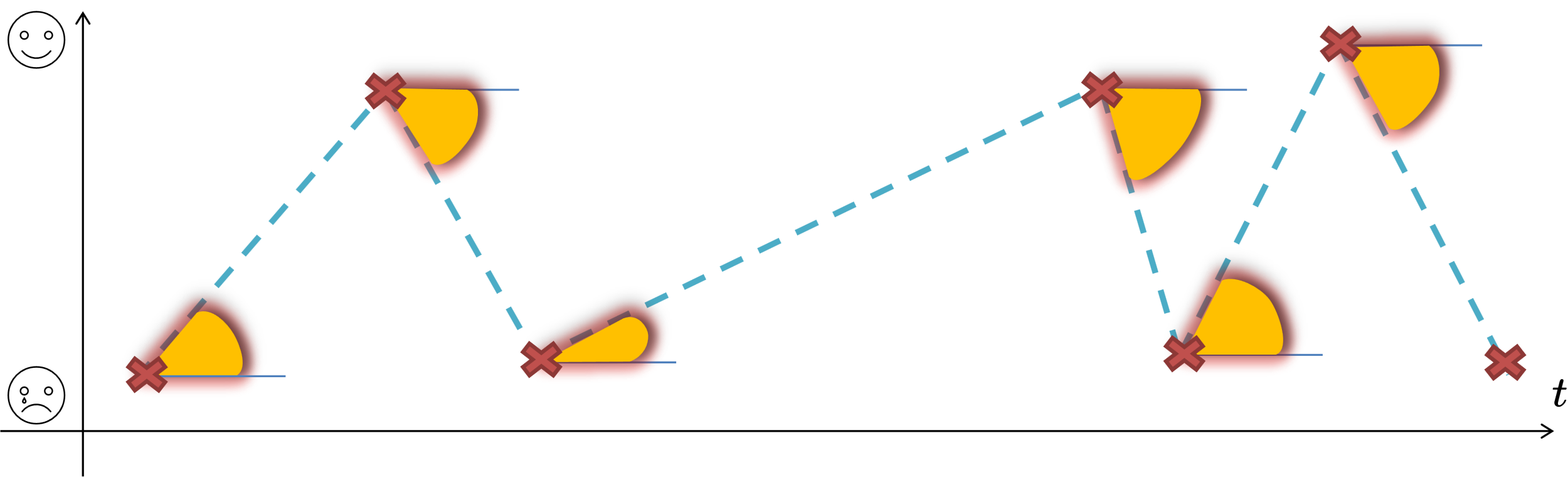


#### Clustering model

With respect to the GMM, an inverse Wishart prior was used for the covariance matrices to thwart numerical instabilities when estimating them. The selected number of degrees of freedom of such a prior was $D+2$, where $D$ is the number of variability dimensions, and the scale matrix was set to diagonal matrix whose main diagonal elements were the overall variance divided by $K^{\frac{1}{D}}$, where $K$ is the number of mixture components. This choice of hyperparameters produces a weak, yet proper prior^2^. The EM algorithm was run for 4 000 random initializations and the one that achieved the highest likelihood was selected. The random initializations for the multidimensional means were obtained by sampling from a (non-negative) gamma distribution whose mean and standard deviation were set as those for the corresponding dimension, and the initialized covariance matrices were obtained from a Wishart distribution that was used to obtain positive definite matrices around the empirical covariance matrix.

#### Random forest

Let $M$ be the number of variables to be evaluated at each step of the forward selection, $\text{max}(\cdot)$ be a function that selects the greatest element of a set, and $\text{floor}(\cdot)$ be a function that rounds toward negative infinity, then, the hyperparameter search space of the random forest was: integers log-scaled in $[10, 500]$, $[1, \text{max}(2,\text{floor}(N/2))]$ and $[1, \text{max}(2,N-1)]$ for the number of decision trees, the minimum number of samples in a leaf, the maximum number of splits for each tree, respectively; and integers from 1 up to $M$ for the number of clinical features to randomly sample to be considered for every split. Each tree of the random forest was built using the Gini's diversity index to find the optimal splits. Let $N$ be the number of patients, then, each bootstrap sample was obtained by sampling $N$ out of the $N$ patients with replacement from the whole dataset, thereby omitting, on average, roughly 36.8% of the patients for each decision tree.

Surrogate splits consist in ranking the clinical features based on their predictive measure of association, viz. their ability to send the patients to the left and right nodes for each decision split as close to what the optimal feature does.

#### Bayesian optimization

Bayesian optimization consists in creating a Gaussian process that models how the random forest would perform for different sets of hyperparameters, in such a way that at every iteration of the Bayesian optimization the best candidate is evaluated. Every evaluated hyperparameter set is also used to refine the Gaussian process. To ensure that the search of the optimal parameter set does not get stranded around some promising areas in the search space (in other words, to balance exploration and exploitation), the Gaussian process modified its kernel function if it determined that was overexploiting an area^3^. The optimized hyperparameters of the random forest using this procedure are shown in Table S2.

**Table S2.** Optimized hyperparameter values of the random forest by Bayesian optimization.

| **Hyperparameter** | **Optimized value** |
| --- | --- |
| Number of decision trees | 20 |
| Minimum number of observations in a leaf | 1 |
| Maximum number of splits for each tree | 94 |
| Number of clinical variables to randomly sample to be considered for every split | 5 |

## Details of the patient selection process

In this paper, a total of 275 of 419 patients from the Smartcrises study^4^ were selected for the analysis. As stated in the methods, there were three requirements: “1) a complete diagnostic assessment with Mini International Neuropsychiatric Interview (MINI) version 7.0.2 is available; 2) the age and gender of the patients are known; and 3) the EMA variability can be computed in at least one domain”. Figure S3 specifies the number of patients that was non included for each of the previous reasons (and their intersections).

**Figure S3.** Venn diagram of the number of patients excluded in the analysis per reason.


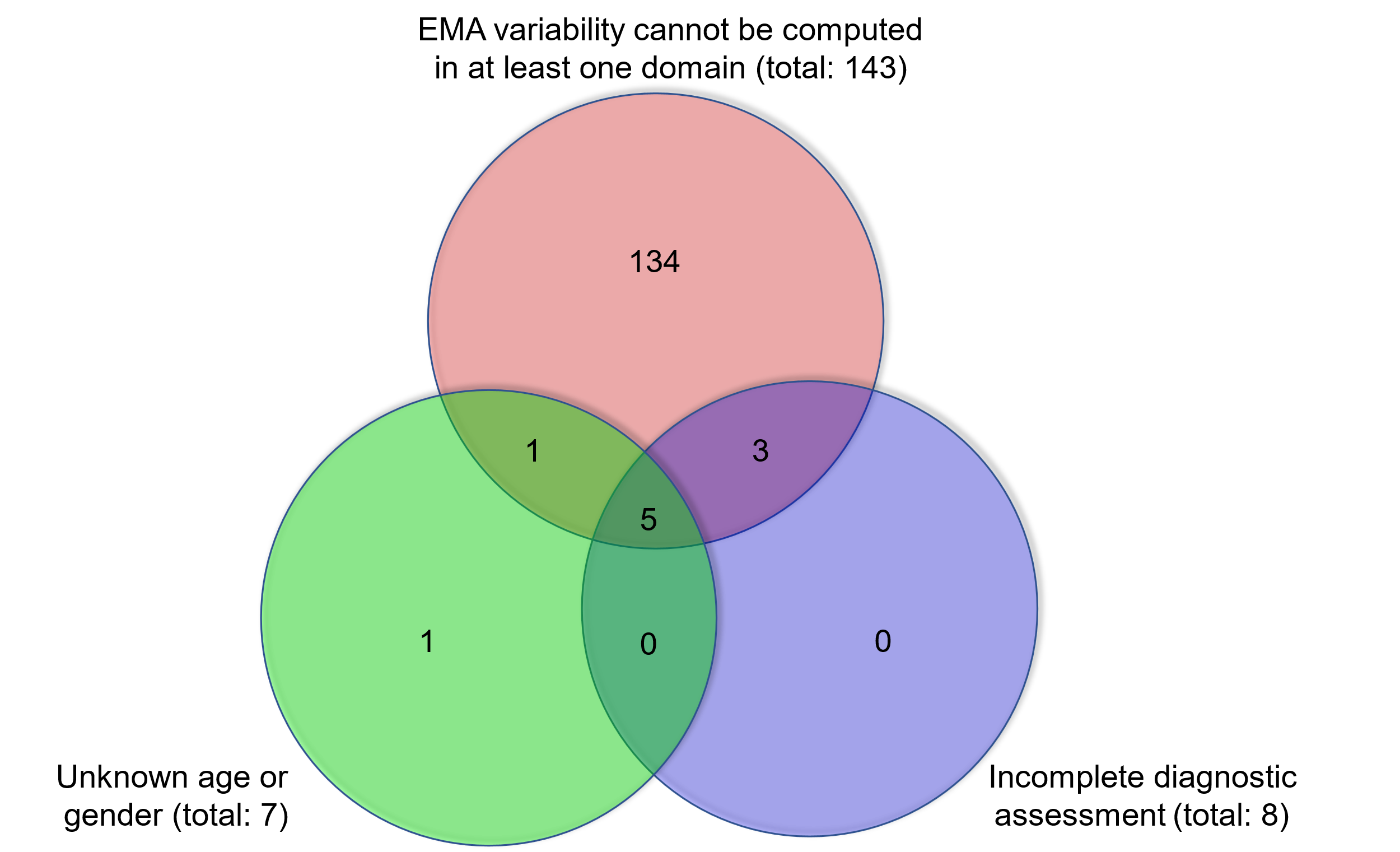


On the other hand, the condition “3) the EMA variability can be computed in at least one domain”, implies that a participant must have answered at least three times the questions of one of the domains. The reason can be understood from Figure S2: we can see that *three* *points* on the plane uniquely define *two* consecutive *slopes*. In that case, the MAD of the absolute value of those two slopes can be computed. Nonetheless, *two* (raw) EMA *observations*, uniquely determine *one* consecutive *slope* between those two points. With just one slope value, there are not enough degrees of freedom to compute the variability of the slopes.

# References

1. Bonilla-Escribano, P., Ramírez, D., Porras-Segovia, A. & Artés-Rodríguez, A. Assessment of variability in irregularly sampled time series: Applications to mental healthcare. *Mathematics* **9**, 71 (2021).

2. Fraley, C. & Raftery, A. E. Bayesian regularization for normal mixture estimation and model-based clustering. *J. Classif.* **24**, 155–181 (2007).

3. Bull, A. D. Convergence rates of efficient global optimization algorithms. *J. Mach. Learn. Res.* **12**, 2879–2904 (2011).

4. Berrouiguet, S. *et al.* Combining mobile-health (mHealth) and artificial intelligence (AI) methods to avoid suicide attempts: The Smartcrises study protocol. *BMC Psychiatry* **19**, 277 (2019).
